# PD-L1 and gastric cancer prognosis: A systematic review and meta-analysis

**DOI:** 10.1371/journal.pone.0182692

**Published:** 2017-08-10

**Authors:** Lihu Gu, Manman Chen, Dongyu Guo, Hepan Zhu, Wenchao Zhang, Junhai Pan, Xin Zhong, Xinlong Li, Haoran Qian, Xianfa Wang

**Affiliations:** 1 Department of General Surgery, Sir Run Run Shaw Hospital, School of Medicine, Zhejiang University, Hangzhou, Zhejiang, China; 2 The Second Clinical College, Zhejiang Chinese Medical University, Hangzhou, Zhejiang, China; 3 Department of Ophthalmology, The First Affiliated Hospital, College of Medicine, Zhejiang University, Hangzhou, Zhejiang, China; National Cancer Center, JAPAN

## Abstract

The expression of Programmed cell Death Ligand 1 (PD-L1) is observed in many malignant tumors and is associated with poor prognosis including Gastric Cancer (GC). The relationship between PD-L1 expression and prognosis, however, is controversial in GC. This paper purports to use a meta-analysis to investigate the relationship between PD-L1 expression and prognosis in GC. For this study, the following databases were searched for articles published from June 2003 until February 2017: PubMed, EBSCO, Web of Science and Cochrane Library. The baseline information extracted were: authors, year of publication, country where the study was performed, study design, sample size, follow-up time, baseline characteristics of the study population, pathologic data, overall survival (OS). A total of 15 eligible studies covering 3291 patients were selected for a meta-analysis based on specified inclusion and exclusion criteria. The analysis showed that the expression level of PD-L1 was associated with the overall survival in GC (Hazard Ratio, HR = 1.46, 95%CI = 1.08–1.98, P = 0.01, random-effect). In addition to the above, subgroup analysis showed that GC patients with deeper tumor infiltration, positive lymph-node metastasis, positive venous invasion, Epstein-Barr virus infection positive (EBV+), Microsatellite Instability (MSI) are more likely to expression PD-L1. The results of this meta-analysis suggest that GC patients, specifically EBV+ and MSI, may be prime candidates for PD-1 directed therapy. These findings support anti-PD-L1/PD-1 antibodies as a kind of immunotherapy which is promising for GC.

## Introduction

Worldwide, gastric cancer (GC) is the fourth most common malignant disease in males (fifth in females) and the third leading cause of cancer mortality in males (fifth in females), especially in Eastern Asia (particularly in Korea, Mongolia, Japan, and China), Central and Eastern Europe, and South America, and lowest in Northern America and most parts of Africa[1]. Developing immunotherapeutic strategies has become a hot area of focus in the treatment of GC. However, the therapeutic efficacy of all immune-checkpoint blockers is not satisfied[2–5]. To date, no phase III clinical trials on the immune-checkpoint blockers have been conducted on GC patients.

Recently, some clinical trials have indicated that monoclonal antibodies that target PD-1 or its receptor PD-L1 prevent the inhibitory effects of PD-1/PD-L1 pathway and enhance T cell functions, leading to impressive outcomes in patients with cancers[6–9]. However, from[10], ‘the predictive effects of PD-L1 in response to PD-1/PD-L1 antibodies in GC are not conclusive and the indication of PD-L1 expression in tumors remains controversial and needs to be further investigated’.

Through a meta-analysis, this review focuses on PD-L1 expression and its association with clinical outcomes in GC. Furthermore, this research attempts to show that the potential of PD-L1 positive patient to obtain optimum treatment benefit, appears promising. It might pinpoint patients most likely to strongly benefit from the inhibition of PD-L1/PD-1 as monotherapy compared to those that may most likely require a different or combinatorial approach in GC.

## Materials and methods

### Search strategy

Studies indexed, from June 2003 until February 2017 were systematically searched in the following databases: PubMed, EBSCO, Web of Science and Cochrane Library. The search terms used were: (“stomach neoplasms” OR “gastric cancer” OR “advanced gastric cancer” OR “gastric carcinoma” OR “stomach cancers”) AND (“PD-1” OR “PD-L1” OR “programmed death 1” OR “programmed death ligand 1” OR “programmed cell death ligand 1” OR “programmed death 1 ligand 1” OR“B7-H1”). Additionally, the reference lists of the selected articles were manually reviewed to obtain other potentially relevant articles. Selected publications were all in the English language.

### Selection criteria

From the potentially relevant articles obtained above, those that indicated correlation between prognosis (including OS and/or clinical significance) and PD-1/PD-L1 in GC were selected.

#### Inclusion criteria

**For inclusion in this meta-analysis**:

1. Articles were limited to those dealing human subjects only.
2. All patients with GC would have been diagnosed by pathological evidence.
3. Expressions of PD-L1 would have been detected by Immunohistochemical (IHC) Assay from gastric carcinoma specimens.
4. All patients had been followed up and results reported.

#### Exclusion criteria

**The following criteria were used to exclude irrelevant papers**:

1. The literature was not the original article (such as meta-analysis, review), or a literature duplication.
2. The object of this study was cellular-based or animal-based.
3. The study also covered other malignancies, or the study did not include the analysis of expressions of PD-L1 in subgroups.

### Data extraction and quality assessment

Data from each included study was independently extracted. The following baseline information was used: authors, year of publication, country where the study was performed, study design, sample size, follow-up time, cut-off criteria for overexpression (the definition of positive PD-L1), baseline characteristics of the study population, pathologic data, overall survival (OS).

This meta-analysis was conducted in accordance with the guidelines of the Preferred Reporting Items for Systematic Review and Meta-Analysis Protocols (PRISMA-P) 2015 statement[11–13]. The quality of the included studies was assessed using the Newcastle-Ottawa Quality Assessment Scale (NOS) checklist, independently by our team authors. This quality-assessment tool focuses on 8 items categorized in three groups (selection, comparability and outcome) with a maximum number of 9 stars. The articles achieving six or more stars were considered high quality[14].

### Statistical analysis

Hazard ratios (HR) including 95% Confidence interval (CI) were used to assess the association between PD-L1 expression level and OS in GC. An observed HR > 1 implied a worse prognostic significance for the group with elevated PD-L1 expression. Conversely, HR < 1 implied a worse prognostic significance for the group with decreased PD-L1 expression. Revman 5.3 Software (RevMan, The Cochrane Collaboration) was used to evaluate heterogeneity between studies by Cochrane Q-test and P-values. Estimates of HR were weighted and pooled using the Mantel-Haenszel random effect model. The Stata 12.0 Software (Stata, College Station) was used to evaluate the sensitivity and publication bias of the studies. Publication bias was evaluated by Begg’s and Egger’s test, P < 0.05 was considered statistically significant. Begg’s and Egger’s test of publication bias was not performed on analysis subgroup with less than 5 studies because of low sensitivity of qualitative and quantitative tests[15].

## Results

### Description of trials included in the meta-analysis

**Fig 1:**
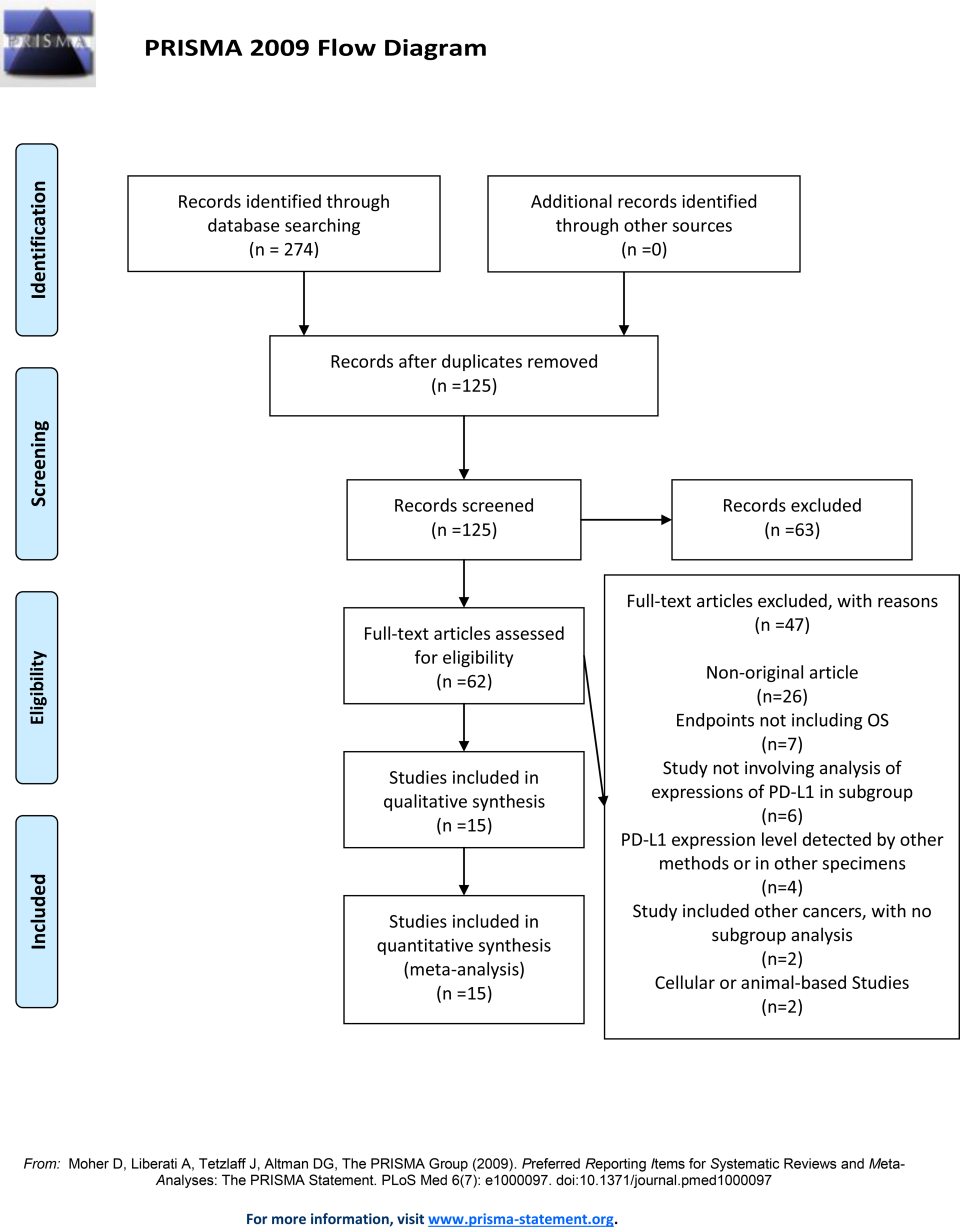
Flow chart of study selection.

The search strategy originally generated 274 relevant clinical studies in English. Of these, 149 were eliminated because of repetition, 63 were excluded based on evaluation of their title or abstract. The remaining 62 articles were scrutinized by a full-text review. Based on the inclusion and exclusion criteria, 15 studies were selected for this meta-analysis. The detailed search and study selection process is shown in Fig 1.

### Study and patient characteristics

The 15 studies published between 2006 until 2016, included 3291 patients at baseline, with a maximum sample size of 451 and a minimum sample size of 96 participants. The majority of the studies were reported in Asia, 7 in China, 4 in Japan, 3 in South Korea, and 1 in Germany. The defining criterion for PD-L1 positive was that PD-L1 expression was observed in tumor cells by IHC. The discrepancies in the percentage of the PD-L1 positive expression patients were due to the variation in the cut-off values with the maximum ratio at 69.40% and the minimum at 14.32%. 1925(58.49%) patients did not undergo radiation or chemotherapy before surgery in 8 studies, others did not report. The characteristics of the included studies have been shown in Table 1.

**Table 1:** Characteristics of studies included in the meta-analysis.

Author, year	Country	No.	Stage	Follow-up, months	PD-L1 (%)	Cut off for positive	Location within tumor cells^	Preoperative Chemoradiotherapy^#^	Surgery	Postoperative adjuvant Chemotherapy No.	Quality Assessment*
Boger *et al*.[16], 2016	Germany	451	I-IV	>20	23.73%	>1%	cytoplasmic	NOT	401 patients received R0 resection and 50 received R1/R2 resection	NA	8
Chang *et al*.[17], 2016	Korea	451	NA	>60	69.40%	NA	cytoplasmic	NA	Gastric resection	NA	7
Dai *et al*.[18], 2016	China	398	I-IV	>61	14.32%	>5%	cytoplasmic	NOT	Gastric resection	275	6
Eto *et al*.[19], 2016	Japan	105	II-III	>34	24.76%	>50%	cytoplasmic and nuclear	NA	Gastric resection	73	8
Geng *et al*.[20], 2015	China	100	I-IV	>60	65.00%	>50%	cytoplasmic and nuclear	NA	Gastric resection	NA	7
Hou *et al*.[21], 2014	China	111	I-IV	NA	63.06%	>10%	NA	NOT	Gastric resection	NA	7
Kang *et al*.[22], 2016	Korea	234	I-III	>65	15.38%	>10%	cytoplasmic	NA	Gastric resection	86	7
Kawazoe *et al*.[23], 2016	Japan	383	III-IV	>75	24.80%	>1%	cytoplasmic	NOT	Gastric resection	261	8
Kim *et al*.[24], 2014	Korea	243	I-III	>74	43.62%	>10%	cytoplasmic	NA	Gastric resection	89	8
Li *et al*.[25], 2016	China	137	I-IV	>17	40.88%	>5%	cytoplasmic	NA	Gastric resection	NA	7
Qing *et al*.[26], 2015	China	107	NA	>42	50.47%	>10%	NA	NOT	Gastric resection	NA	6
Saito *et al*.[27], 2016	Japan	96	NA	NA	34.38%	>5%	cytoplasmic	NA	Gastric resection	NA	6
Tamura *et al*.[28], 2015	Japan	241	I-IV	>60	53.11%	>50%	NA	NOT	Gastric resection	65	8
Wu *et al*.[29], 2006	China	102	NA	>42	42.16%	NA	cytoplasmic and nuclear	NOT	Gastric resection	NA	7
Zhang *et al*.[30], 2015	China	132	II-III	>66	50.76%	NA	cytoplasmic	NOT	40 patients received D1 resection and 92 patients received D2 resection	63	7

^: PD-L1 expression location within the tumor cell as observed by the study

NA: Not available

NOT: No patients underwent pre-operative chemoradiotherapy

*: Quality Assessment based on Newcastle-Ottawa Scale

### Prognosis

**Fig 2:**
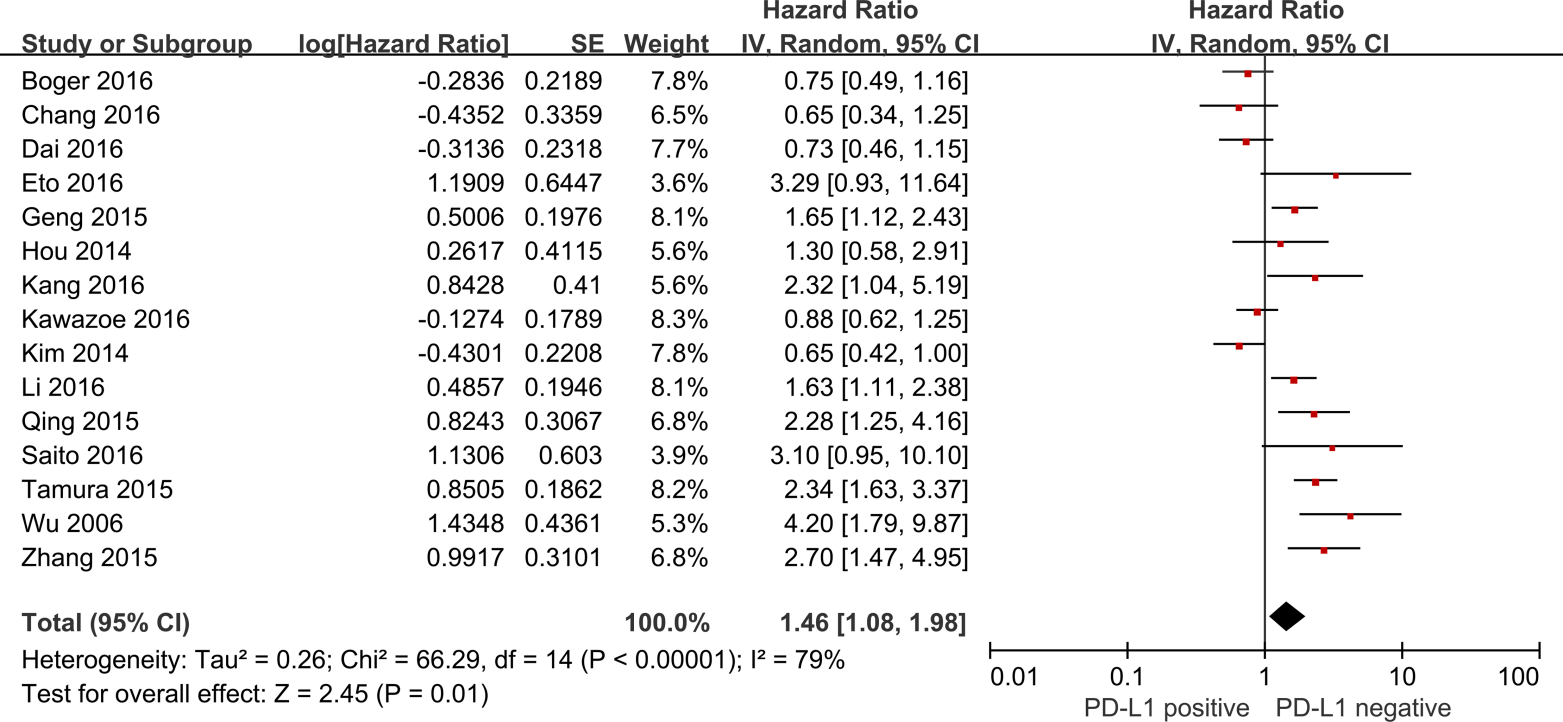
Forest plot describing the association between PD-L1 positive and HR of patients with GC.

A total of 15 studies reported that the expression level of PD-L1 was related to OS. 11 studies indicated that PD-L1 overexpression was associated with poor prognosis of GC, conversely, 3 studies reported that PD-L1 overexpression was associated with better prognosis, and there was no indicated associations 1 study. Analysis showed that the expression level of PD-L1was associated with the OS in GC (HR = 1.46, 95%CI = 1.08–1.98, P = 0.01, random-effect) (Fig 2).

### Correlation of PD-L1 expression with clinicopathological characteristics

As shown in Table 2, a meta-analysis was performed to evaluate the relationship between the PD-L1 expression and clinicopathological characteristics in GC. Results demonstrated that the PD-L1 overexpression was significantly related to depth of infiltration (T-stage), lymph-node metastasis (N-stage), venous invasion, Epstein-Barr virus (EBV) infection, MSI-status. On the contrary, there was no clear relationship with sex, age, tumor site, tumor size, tumor differentiation, Lauren-Classification, TNM stage, lymphatic invasion, neural invasion (S1 Fig). Exploratory subgrouping was performed as per the ethnicity, stages of cancer, location within the tumor cells, pre operative chemoradiotherapy and post surgery adjuvant chemoradiotherapy, follow-up time of more than 5 years and also the PD-L1 positive cut-off value (S2 Fig). The analysis suggested that for the following exploratory subgroup: Asian, stages II-III, cut-off value of more than 50% and also the cytoplasmic and nuclear PD-L1 expression location within the tumor cell, the P values demonstrated a positive association with the OS in GC (Table 3).

**Table 2:** Correlation of PD-L1 expression with clinicopathological characteristics and Begg’s and Egger’s test in subgroup analysis.

Subgroup analysis	No. of studies	No. of patients	Experimental group: positive/total	Control group: positive/total	OR	95% CI	P value	Heterogeneity (I^2^)	Begg's test (P value)	Egger's test (P value)
**Gender**	14	2958	Male 681/2073 (32.85%)	Female 282/885 (31.86%)	1.15	0.96–1.38	0.12	0%	0.511	0.572
**Age1**	4	707	<60years 79/302 (26.16%)	≥60years 140/405 (34.57%)	0.74	0.49–1.11	0.15	18%	-	-
**Age2**	2	938	<65years 237/515 (46.02%)	≥65years 187/423 (44.21%)	0.65	0.39–1.08	0.10	63%	-	-
**Age3**	2	484	<70years 154/327 (47.09%)	≥70years 80/157(50.96%)	0.94	0.60–1.48	0.80	22%	-	-
**Tumor site**	10	2060	Proximal tumor 358/1106 (32.37%)	Distal tumor 316/954(33.12%)	1.05	0.74–1.47	0.79	59%	0.858	0.882
**Tumor size**	5	509	<5cm 142/287 (49.48%)	≥5cm 130/222(58.56%)	0.67	0.42–1.05	0.08	36%	1.000	0.991
**Tumor differentiation**	12	2709	well and moderately differentiation 437/1107 (39.48%)	poorly differentiation 639/1602(39.89%)	0.93	0.52–1.65	0.80	89%	0.304	0.632
**Lauren Classification**	4	1101	Intestinal 175/622 (28.14%)	Diffuse 90/479(18.79%)	1.58	0.54–4.64	0.40	90%	-	-
**Depth of infiltration**	10	2438	T1/T2 stage 291/858 (33.92%)	T3/T4 stage 657/1580(41.58%)	0.47	0.24–0.93	0.03	89%	0.592	0.756
**Lymph-node metastasis**	12	2633	N- 338/948(35.65%)	N+ 708/1685(42.02%)	0.54	0.31–0.95	0.03	86%	0.193	0.939
**TNM stage**	9	1926	I/IIstage 306/852(35.92%)	III/IV stage 380/1074(35.38%)	0.72	0.40–1.28	0.26	85%	0.602	0.450
**Lymphatic invasion**	7	1857	lymphatic invasion- 342/864(39.58%)	lymphatic invasion+ 411/993(41.39%)	0.66	0.32–1.36	0.26	88%	0.072	0.050
**Venous invasion**	6	1623	Venous invasion- 507/974(52.05%)	Venous invasion+ 210/649(32.36%)	0.52	0.36–0.74	0.0003	5%	0.707	0.806
**Neural invasion**	4	1326	Neural invasion- 337/853(39.51%)	Neural invasion+ 175/473(37.00%)	0.74	0.25–2.20	0.58	92%	-	-
**E-B virus infection**	4	1307	E-B virus + 94/171(54.97%)	E-B virus– 223/1136(19.63)	15.50	4.17–57.62	<0.0001	77%	-	-
**MSI-status**	2	937	MSI 36/61(59.02%)	MSS 176/876(20.09%)	6.09	2.44–15.25	0.0001	62%	-	-

**Table 3:** Exploratory subgrouping analysis of heterogeneity.

		No. of studies	HR	95% CI	P value	Heterogeneity (I^2^)
**Asian**		14	1.54	1.13–2.11	**0.007**	78%
**Stages**	**I-IV**	6	1.30	0.87–1.94	0.20	80%
	**II-III**	2	2.80	1.62–4.84	**0.0002**	0%
	**I-III**	2	1.17	0.34–4.07	0.80	87%
**Follow-up more than 5 years**		8	1.24	0.82–1.88	0.30	84%
**Cut-off value**	**>1%**	2	0.83	0.63–1.08	0.17	0%
	**>5%**	3	1.36	0.66–2.78	0.40	79%
	**>10%**	4	1.41	0.70–2.85	0.34	80%
	**>50%**	3	2.03	1.53–2.71	**<0.00001**	11%
**Location within tumor cells**	**cytoplasmic**	9	1.12	0.79–1.60	0.52	76%
	**cytoplasmic and nuclear**	3	2.50	1.28–4.90	**0.007**	55%
**No preoperative Chemoradiotherapy**		8	1.51	0.97–2.35	0.07	84%
**Postoperative adjuvant Chemotherapy**		7	1.42	0.86–2.33	0.17	85%

### Publication bias and sensitivity analysis

**Fig 3:**
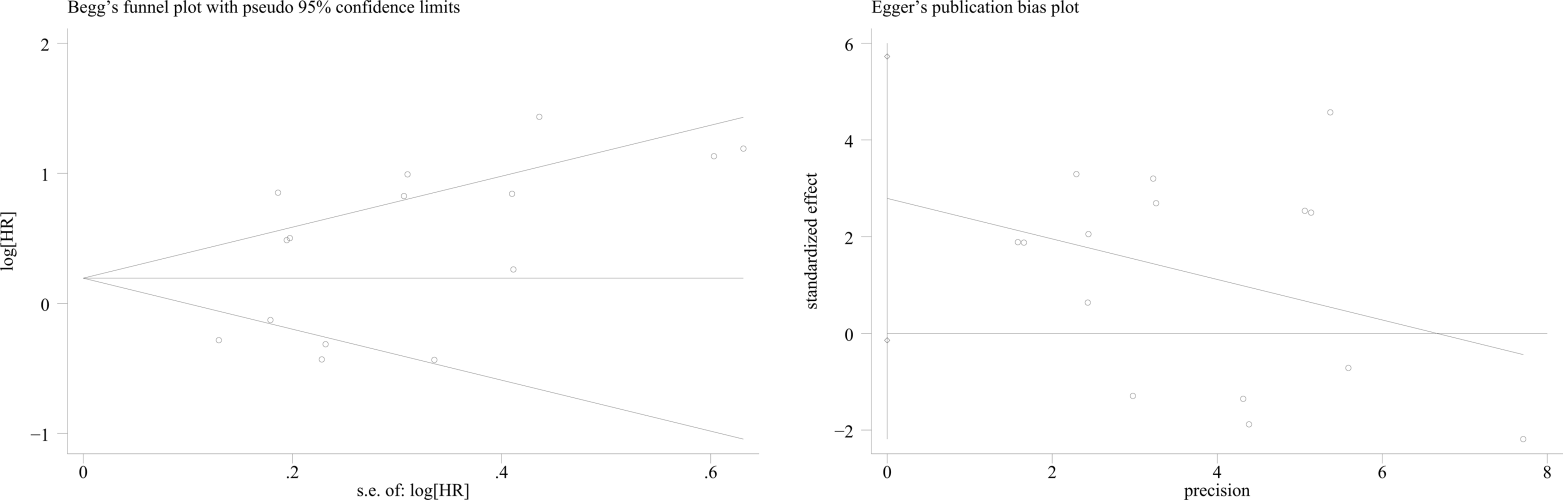
Begg’s and Egger’s funnel plot with 95% CI for OS publication bias testing.

Begg’s and Egger’s test was used to evaluate the publication bias, respectively. In this meta-analysis, Begg’s and Egger’s test indicated no publication bias among included articles regarding the HR and OS (Begg’s test: P = 0.276 and Egger’s test P = 0.061) (Fig 3). There was no publication bias in subgroups analysis, as indicated by all the P values being greater than 0.05 (Table 2 and S3 Fig). Stata12.0 software was used to perform sensitivity analysis to assess whether the individual studies do not affect the overall results. The results indicated that individual study had little influence on the final results, and demonstrated that the analysis was relatively stable and credible (S4 Fig).

## Discussion

Based on the theory of The Cancer-Immunity Cycle, T cells have been the major focus of efforts to therapeutically manipulate endogenous anti-tumor immunity. However, for various reasons (factors in the tumor microenvironment might suppress effector T (T_eff_) cells that are produced), the activated T_eff_ cells can’t specifically recognize and bind to cancer cells, T cells therefore cannot kill their target cancer cells. In other words, some molecules, namely factors, may act to modulate activated anti-tumor T cell, such as PD-L1[31].

PD-L1(B7-H1) is a B7-family member that has been ascribed as regulating T cell functions through the engagement with PD-1, a CD28family member receptor[32, 33]. Recent clinical trials have shown that anti-PD-L1/PD-1 antibodies produced both durable tumor regression and prolonged disease stabilization in patients with non-small-cell lung cancer[7], melanoma[6], renal-cell cancer[8], and pancreatic cancer[9]. Chen *et al*.[31] described the anti-cancer mechanism as when the antibodies block the PD-L1/PD-1 interaction, T_eff_ cells can restore their anti-cancer function. Blank *et al*.[34] have demonstrated that blocking PD-L1 can improve immune functions of tumor-specific T_eff_ cells when interacting with their target tumor cells in vitro.

However, the relationship between PD-L1 expression and prognosis is still subject to much controversy in GC. Boger *et al*.[16] reported that patients with PD-L1 positive tumor cells had a significantly improved prognosis, conversely, Chang *et al*.[17] showed that high PD-L1 expression was a significant adverse prognostic factor. In addition, Kawazoe *et al*.[23] indicated that PD-L1 was not a prognostic factor.

Recently, a few meta-analyses have shown a correlation between PD-L1 and prognosis in GC; Wu *et al*.[35], Xu *et al*.[36], and Liu *et al*.[37] demonstrated that PD-L1 overexpression was a worse prognostic factor in GC. However, their analyses covered small number of studies (3, 5 and 8 studies respectively) and also lacked further subgroup analysis. Zhang *et al*.[38] reported that PD-L1 positive was a risk factor for OS but it did not analyze the association between PD-L1 positive and Lauren-Classification, lymphatic, venous, neural invasion. To point out, sample patients are more from the East Asian community. Results may not apply in general to all human types, as it is known that reactions to medicine are not the same in general. In our study, the relation between the PD-L1 expression and a specific molecular subgroup such as E-B virus infection GC and MSI were analyzed.

Consistently with the experimental results of Ma *et al*.[39] and Derks *et al*.[40], this meta-analysis also demonstrated that EBV+ Gastric Cancer and MSI tend to show positive PD-L1 expression. The results of this meta-analysis, covering 3291 patients, showed that PD-L1 overexpression is a significant adverse prognostic factor. This finding fits the theory of the Cancer-Immunity Cycl[31]. In this theory, dead cancer cells release antigens, which are then captured by dendritic cells(DCs) which in turn process and present them. The latter prime and activate the T_eff_ cells responses against the cancer-specific antigens. The activated T_eff_ cells, then, traffic to and infiltrate the tumor bed, specifically recognize and bind to cancer cells and kill their target cancer cell. In cancer patients, the Cancer-Immunity Cycle does not perform optimally, one of the reasons (or most importantly) may be that some immune rheostat factors (such as PD-L1/PD-1) in the tumor microenvironment might be suppressing those T_eff_ cells that are produced. Our findings concur with results of several clinic trials about anti-PD-L1/PD-1 antibodies, which supported the importance of the PD-L1/PD-1 pathway in GC, and demonstrated that anti-PD-L1/PD-1 antibodies can be safely given to patients and provided sustained anti-cancer activity.

As mentioned by[41]: ‘Regulatory T (T_reg_) cells are the main mediators of peripheral tolerance. They actively suppress T_eff_ cells and inhibit immune-mediated tissue damage’. T_reg_ cells can be divided into naturally occurring T_reg_ (nT_reg_) and induced T_reg_(iT_reg_) cells[42]. PD-L1 is highly expressed on T _reg_ cells and can make iT_reg_ cells express some molecules (such as CD25, CTLA-4), which can potentially suppress T_eff_ cells, owing to: [(1) PD-L1 can induce the development of functional fork head box p3^+^ (Foxp3^+^) iT_reg_ cells, (2)PD-L1 enhances and maintains Foxp3 expression on iT_reg_ cell and augments suppression at low T_reg_/T _eff_ cell ratios. In addition, the TGF-βcan synergize the phenomenon.] Increased PD-L1 expression by tumor cells may induce and maintain iT_reg_ cells in the periphery, thereby increasing the suppression of anti-tumor T cell responses and allowing tumor progression[43].

In effect, this research paper tries to evaluate the association between the PD-L1 overexpression and the clinical pathological features. From this meta-analysis, gastric cancer patients with deeper tumor infiltration, positive lymph-node metastasis, positive venous invasion, E-B virus infection positive, MSI are more likely to express PD-L1. The results of subgroup analysis tend to support the idea that patients with positive PD-L1 have unfavorable prognosis.

A recent study[44] aiming to perform a comprehensive molecular profiling found that key driver genes were enriched in a specific molecular subgroup: [(1) Epstein-Barr Virus positive (EBV+) GC, (2) Microsatellite Instability (MSI), (3) Chromosomal Instability (CIN) or (4) Genomically stable (GS) tumors]. EBV+ GC and MSI GC have rich lymphocytic infiltration in tumor stroma and thus can be classified as gastric carcinoma with prominent lymphoid stroma (medullary carcinoma). The lymphoid stroma in these tumors has high number of CD8 T cells, capable of mounting a robust antitumor inflammatory response. Besides, the positive PD-L1 expression was associated with a concomitant, significant increase in the number of CD8 T cells at tumor invasive front[39].

Beyond the differential presence of PD-L1+ cells in EBV+ and EBV- GC, Derks *et al*.[40] observed a difference in infiltration pattern of PD-L1 positive immune cells; while PD-L1+ immune cells were able to infiltrate the center of EBV+ and MSI GC, in EBV- and MSS GC PD-L1+ immune cells stayed mainly at the invasive margin. Further analysis showed that compared to EBV- GC, EBV+ GCs indeed have strong enrichment of IFN-γ response genes. The combination of PD-L1 positivity and enrichment for an IFN-γ signature in EBV+ GCs suggests the potential for PD-L1 expression and activation of the PD-1 pathway to be a critical mechanism in these tumors to control an antecedent cytotoxic anti-tumor immune response, which increases the likelihood of response to PD-1 blockade in this GC subtype. Interestingly, besides EBV+ GCs, MSI GCs have high IFN-γ response gene expression, perhaps reflecting the large lymphocyte infiltrate that is typical for mismatch-repair deficient cancers with a high mutational load.

To the best of our knowledge, it is the first meta-analysis to investigate the association between PD-L1 expression and E-B virus infection and MSI in GC. This study shows that the expression level of PD-L1 is higher in EBV+ GC and MSI than in EBV- GC and Microsatellite stability (MSS). The result suggests that specifically EBV+ GC and MSI may be prime candidates for PD-1directed therapy.

### Limitations

The limitations of this study should be stressed on. The heterogeneity among included studies cannot be ignored. We were unable to control for factors such as environmental conditions, racial differences, socioeconomic situation, postoperative treatment, follow-up, all of which are known to influence the OS. Besides, these studies have used a number of monoclonal and polyclonal PD-L1 antibodies for immunohistochemistry and a variety of different scoring schemes/criteria to define positive PD-L1 expression. For example, Eto *et al*.[19] and Geng *et al*.[20] reported that when the percentage of PD-L1positivecancer cells was greater than 50%, which was the definition of PD-L1 positive, while, Kawazoe *et al*.[23] and Boger *et al*.[16] considered that PD-L1-positive cases on tumor cells were defined by the presence of at least 1% of tumor cells with membrane staining. The different ratio of the patient with PD-L1 overexpression ranged from 14%[18] to 69%[17].

Publication bias is another possible reason, wherein studies showing that PD-L1 was not a prognostic factor could be less likely to be published than studies showing that PD-L1 was a prognostic factor. Then the included studies were only English researches, no other languages. Beyond publication bias, the patients covered in this study were strictly from the countries where the studies were made. The conclusion would hence apply to those countries only unless the study is extended to other countries representatively. Perhaps the racial and social factors were also at play here as it has also been observed in other fields other than medicine. Their effects could have been cofounded with those being measured here. More representative samples of patient studies could be needed for stronger conclusion. Also, grey literature was not accounted for in this study as only studies published in the English language were chosen[45, 46].

## Conclusion

This meta-analysis was to firstly evaluate the association between the PD-L1 expression and a specific molecular subgroup (EBV+ and MSI) in GC. Furthermore, gastric cancer patient with EBV+ and MSI tend to show PD-L1 expression, which demonstrated that specifically EBV+ GC and MSI may be prime candidates for PD-L1 directed therapy. However, further large-scale and comprehensive researches are needed to support our results and conclusion.

## Supporting information

S1 Fig Forest plots evaluating the association between PD-L1 positive and OS of clinicopathological characteristics. (A) sex, (B) age, (C) tumor site, (D) tumor size, (E) tumor differentiation, (F) Lauren Classification, (G) depth of infiltration,(H) lymph-node metastasis, (I) TNM stage, (J) lymphatic invasion, (K) venous invasion, (L) neural invasion, (M) E-B virus infection, (N)MSI-status.
(TIF)

S2 Fig Exploratory subgrouping analysis of heterogeneity. (A) Asian, (B-1) stages I-IV, (B-2) stages II-III, (B-3) stages I-III, (C) follow-up more than 5 years, (D-1) cut-off value >1%, (D-2) cut-off value >5%, (D-3) cut-off value >10%, (D-4) cut-off value >50%, (E-1) location within tumor cells were cytoplasmic, (E-2) location within tumor cells were cytoplasmic and nuclear, (F) no patients underwent pre-operative chemoradiotherapy, (G) patients underwent postoperative adjuvant Chemotherapy.
(TIF)

S3 Fig Begg’s and Egger’s test for publication bias in subgroup analysis. (A) sex, (B) tumor site, (C) tumor size, (D) tumor differentiation, (E) depth of infiltration, (F) lymph-node metastasis, (G) TNM stage, (H) lymphatic invasion, (I) venous invasion.
(TIF)

S4 Fig Sensitivity analyses of the studies in subgroup analysis. (A) sex, (B) tumor site, (C) tumor size, (D) tumor differentiation, (E) depth of infiltration, (F) lymph-node metastasis, (G) TNM stage, (H) lymphatic invasion, (I) venous invasion,(J)OS.
(TIF)

S1 Table PRISMA 2009 checklist. (DOC)
